# The Non-Genomic Actions of Vitamin D

**DOI:** 10.3390/nu8030135

**Published:** 2016-03-02

**Authors:** Charles S Hii, Antonio Ferrante

**Affiliations:** 1 Department of Immunopathology, SA Pathology at the Women’s and Children’s Hospital, 72 King William Road, Adelaide, SA 5006, Australia; antonio.ferrante@adelaide.edu.au; 2 Robinson Research Institute, University of Adelaide, Ground Floor, Norwich Centre, 55 King William Road, Adelaide, SA 5006, Australia; 3 Molecular and Cellular Biology, School of Biological Sciences, University of Adelaide, Adelaide, SA 5005, Australia

**Keywords:** vitamin D, vitamin D receptor, signalling molecules, MAP kinases, protein kinase C, vitamin D response element

## Abstract

Since its discovery in 1920, a great deal of effort has gone into investigating the physiological actions of vitamin D and the impact its deficiency has on human health. Despite this intense interest, there is still disagreement on what constitutes the lower boundary of adequacy and on the Recommended Dietary Allowance. There has also been a major push to elucidate the biochemistry of vitamin D, its metabolic pathways and the mechanisms that mediate its action. Originally thought to act by altering the expression of target genes, it was realized in the mid-1980s that some of the actions of vitamin D were too rapid to be accounted for by changes at the genomic level. These rapid non-genomic actions have attracted as much interest as the genomic actions and they have spawned additional questions in an already busy field. This mini-review attempts to summarise the *in vitro* and *in vivo* work that has been conducted to characterise the rapid non-genomic actions, the mechanisms that give rise to these properties and the roles that these play in the overall action of vitamin D at the cellular level. Understanding the effects of vitamin D at the cellular level should enable the design of elegant human studies to extract the full potential of vitamin D to benefit human health.

## 1. Introduction

Vitamin D came to prominence when it was found that its deficiency underlies the pathogenesis of rickets in children and osteomalacia in adults. Subsequent investigations revealed that its deficiency and insufficiency, measured as serum 25-hydroxyvitamin D of <29 ng/mL, are associated with osteoporosis and nonskeletal diseases such as autoimmune diseases, inflammatory bowel disease, bacterial and viral infections, cardiovascular disease, cancer and neurocognitive disorders (reviewed in [1]). Low vitamin D levels are also associated with increased risk of all-cause mortality [1].

It has been estimated that vitamin D deficiency affects approximately one billion people in the world [2]. The major cause is thought to be our predominantly urbanized indoor lifestyle since our main source of vitamin D is its precursor that is synthesised in the skin upon exposure to the sun or UVB irradiation (see below). In order to increase the plasma levels of vitamin D, many have advocated diet supplementation. Nutritionally, vitamin D can be obtained from cod liver oil, fatty fish, eggs and vitamin D-fortified supplements. Although there is consensus for a need to maintain an adequate body level of vitamin D, there is still disagreement on what constitutes the lower boundary of adequacy and what constitutes adequacy or a healthy level amongst the different learned societies and reference laboratories [1,3]. In order to increase the plasma levels of vitamin D, apart from sun exposure, many have advocated diet supplementation and again, there is disagreement on the Recommended Dietary Allowance (RDA) for vitamin D. To exacerbate these issues, it was recently reported that the Institute of Medicine had made a serious calculation error of the RDA such that these values are greatly underestimated [4,5]. Furthermore, many factors, including age, body mass index, ethnicity, calcium intake, oestrogen use, genetics, dietary fat content and composition, and duration of supplementation affect the degree to which a given dose of vitamin D supplementation will raise serum 25-hydroxyvitamin D concentrations [3]. At the other end of the spectrum, too much vitamin D in the body may be harmful. For example, although refuted, some reports have stated that high vitamin D levels are associated with increased risk of all-cause mortality [1]. However, other factors may muddle the picture in situations where normal-high serum vitamin D levels have been associated with adverse outcomes. For example, it has recently been reported that whereas African-American men who have low vitamin D levels and are therefore at a higher risk of prostate cancer may benefit from higher vitamin D intake, supplementation with dietary flavonoids, such as quercetin, in those with normal vitamin D levels may increase their risk of prostate cancer [6]. Thus, much remains to be resolved nutritionally in order to fully exploit the benefits of vitamin D and its metabolites.

The question of how vitamin D acts at the cellular level has intrigued many and this has enabled the elucidation of the biochemistry of vitamin D and its metabolites, and an understanding of its effects on target cells and its mode of action. Vitamin D is derived from 7-dehydrocholesterol, which is converted in the skin under Ultraviolet light band B (UVB) to vitamin D_3_ (cholecalciferol), an inactive precursor. This is then converted to the biologically active vitamin D_3_ metabolite, 1,25-dihydroxyvitamin D_3_ (1,25D), also known as calcitriol. This secosteroid hormone plays important roles in a range of physiological processes including Ca^2+^ and phosphate homeostasis and bone remodelling, cellular proliferation and differentiation and immune regulation [7,8,9,10] Consequently, achieving an adequate level of vitamin D in the body is important in preventing the development of the conditions discussed above. The levels of 1,25D are tightly regulated by the mitochondrial hydroxylases, cytochromes P450C1α (CYP27B1) and P450C24 (CYP24) that catalyse the bioactivation and degradation of vitamin D_3_ metabolites, respectively [11].

## 2. Vitamin D and Non-Genomic Actions

**Figure 1:**
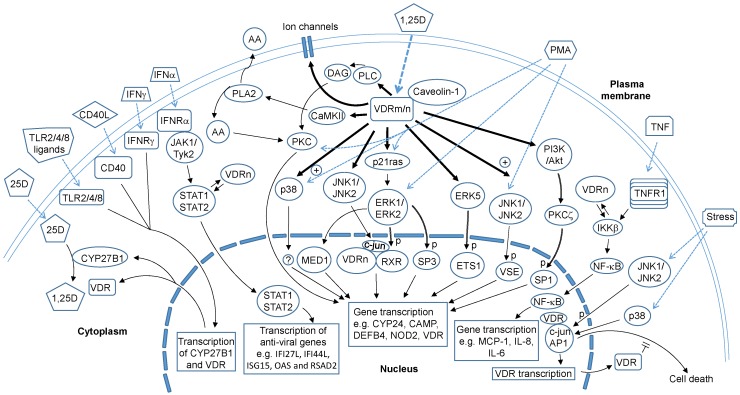
The non-genomic actions of 1,25D. 1,25D acts via both genomic and non-genomic actions to regulate the expression of vitamin D responsive genes such as CY24 and those that promote anti-microbial defence. The non-genomic mechanisms, downstream of VDRn and/or VRDm complexed to caveolin1, include the activation of intracellular signalling molecules, such as PKC, PI3K, MAP kinases, CaMKII and PLA_2_. The range of signalling molecules being activated is related to the cell-type and status of cell maturation. Targets of these kinases include transcription factors, e.g., SP1, SP3 and RXR that bind to response elements on the promoters of vitamin D-responsive genes. Another non-genomic action of 1,25D involves the regulation of VDR binding to target proteins such as STAT1 and IKKβ that enables the cross modulation by 1,25D of gene expression mediated by non-vitamin D ligands, including IFN-α and TNF-α. This mechanism provides an avenue for 1,25D to directly regulate immune responses and anti-viral actions of immune and non-immune cells. Ligation of CD40, IFN-γ receptor and TLR2/4/8 causes an upregulation of VDR and CYP27B1 expression and this facilitates an autocrine mechanism that enables the increased availability of 1,25D and VDR to cooperate with these immune receptors for various responses, including anti-microbial activity while modulating the expression of cytokines, chemokines and type 1 interferons. The VDR, independently of 1,25D, can also exhibits a non-classical action that involves its interaction with proteins such as transcription factors or kinases to modulate cellular responses, including immune/antiviral response and stress-induced cell death. Bold arrows denote direct actions of 1,25D. Dotted arrows denote ligand binding to their receptors. Reversible arrows indicate protein-protein interaction. Arrows with double lines indicate kinase-substrate relationship. Abbreviations: 25D: 25-hydroxyvitamin D; 1,25D: 1,25 dihydroxyvitamin D; VDRm/n: membrane or nuclear vitamin D receptor; CYP27B1 and CYP24: cytochromes P450C1α and P450C24; PLA2: phospholipase A2; CaMKII: Calcium/calmodulin protein kinase II; AA: arachidonic acid; DAG: diacylglycerol; PLC: phospholipase C; IFN: interferon; TLR: Toll-like receptor; PMA: phorbol 12-myristate 13-acetate; PKC: protein kinase C; PI3K: phosphatidylinositol 3-kinase; Akt: protein kinase B; IKK: I-κB kinase; MED1; Mediator Complex Subunit 1; RXR: retinoic acid X receptor; Specificity Protein; ETS 1: Avian Erythroblastosis Virus E26 Oncogene Homolog-1; VSE: Vitamin D stimulatory element; STAT: Signal Transducer and Activator of Transcription; JAK: Janus kinase; Tyk: Tyrosine kinase; ERK: Extracellular signal Regulated protein Kinase; JNK: c-Jun N-terminal Kinase; MCP-1: Monocyte chemoattractant protein 1; CAMP: Cathelicidin Antimicrobial Peptide; IFI27L: Interferon, Alpha-Inducible Protein 27 like; IFI44L: Interferon-Induced Protein 44-Like; ISG15: Interferon-Stimulated Protein, 15 KDa; OAS: 2′-5′-Oligoadenylate Synthetase; RSAD2: Radical S-Adenosyl Methionine Domain Containing 2; “p” denotes phosphorylation; “+” denotes enhancement.

Classically, the effects of 1,25D are thought to be mediated by its interaction with a nuclear vitamin D receptor (VDRn) (Figure 1), a member of the nuclear receptor superfamily of ligand-activated transcription factors [12]. Liganded VDRn forms a heterodimeric complex with Retinoid-X-Receptor (RXR) and either upregulates or downregulates the expression of target genes through binding to promoter sequences termed a vitamin D_3_ response element. It is recognised that 1,25D also exerts non-genomic actions that are manifested, in the main, as the activation of signalling molecules, such as phospholipase C and phospholipase A_2_ (PLA_2_), phosphatidylinositol-3 kinase (PI3K) and p21ras, and the rapid generation of second messengers (Ca^2+^, cyclic AMP, fatty acids and 3-phosphoinositides such as phosphatidylinositol 3,4,5 trisphosphate), accompanied by the activation of protein kinases, such as protein kinase A, src, mitogen-activated protein (MAP) kinases, protein kinase C (PKC) and Ca^2+^-calmodulin kinase II [12,13,14,15,16] (Figure 1). The non-genomic actions also include the opening of Ca^2+^ and Cl^−^ channels [17]. Furthermore, it has also been recognized that the VDR can exert non-classical actions (Figure 1). These are discussed below.

## 3. Vitamin D Receptors

Much effort has been expended to address how the non-genomic actions are mediated, what receptor(s) is (are) involved, whether the VDRn plays a role and how important the non-genomic actions are to the overall response to 1,25D. A distinct membrane VDRm was originally proposed by Norman and colleagues on the basis that some vitamin D analogues that were incapable of binding VDRn were able to initiate the rapid responses of 1,25D [17,18]. The authors identified a specific binding protein for 1,25D in the basal-lateral membranes of chick intestinal epithelium that regulates Ca^2+^ transport across such membranes [19]. This receptor was named membrane-associated rapid response steroid (MARRS) binding protein and it was later identified to be a protein known by several different names: thioredoxin-like protein or GRP58 (for glucose responsive protein, 58 kDa), endoplasmic reticulum protein 57/60 kDa (ERp57 or ERp60) and Protein Disulfide Isomerase Family A, Member 3 (Pdia3) [12,13]. This VDRm can be visualised around the cell surface and the perinuclear area but not in the nucleus. At the plasma membrane, the receptor is localised to the caveolae where it binds caveolin-1 and phospholipase A_2_ [20,21].

## 4. VDRn and VDRm in Intracellular Signalling and Mechanism of Action

While the existence of a VDRm and its role in the non-genomic actions of 1,25D are well-accepted, there has been some debate as to whether VDRn participates in the non-genomic actions of 1,25D. Early work by Boyan and colleagues has led to the conclusion that VDRn plays no role in the activation of PKC as by 1,25D well as some of the genomic actions of 1,25D, at least in murine growth zone costochondral chondrocytes [22]. Thus, whereas 1,25D failed to inhibit the proliferation of VDRn-/- cells, in contrast to its anti-proliferative action in wild-type cells, the hormone-mediated activation of PKC and stimulation of proteoglycan synthesis appeared unaffected [22]. Employing RNA interference (RNAi) to knock down either VDRn or Pdia3, this group has also reported that, in contrast to the lack of dependency of PKC activity on VDRn, 1,25D-mediated activation of src and phospholipase A_2_ requires both the VDRm (Pdia3) and VDRn in MC3T3-E1 osteoblasts [21]. A requirement for VDRn in the activation of signalling molecules such as the MAP kinaseas and PI3K has also been investigated. Yamauchi *et al*. [23] reported that RNAi-mediated VDRn knockdown in Caco-2/TC7 cells had no effect on the activation of ERK1/ERK2 in response to 1,25D. However, examination of their data reveals variable degrees of ERK1/ERK2 activation in these VDRn-knockdown cells, making it difficult to draw conclusions. In contrast, studies using C2C12 murine myoblasts in which VDRn was knocked down by RNAi support the argument that VDRn is involved in mediating 1,25D-stimulated activation of p38 and ERK1/ERK2, src and PI3K/Akt [24]. These observations suggest that there may be a selective usage of VDRn for distinct non-genomic/genomic signalling pathways. Nevertheless, caution may need to be exercised in drawing the conclusion that VDRn is not required for PKC activation for two reasons. The early work on PKC activation measured the specific activity of soluble PKC in cell lysates relative to total cell lysate protein, rather than to total PKC protein [22]. More importantly, the need for PKC translocation to membrane/cytoskeletal elements where the natural substrates reside [25] was not taken into consideration using the assay. A second reason is the recent observation from the same group of a substantial (>50%) reduction in 1,25D-stimulated PKC activity in MC3T3-E1 osteoblasts that had had their VDRn knocked down by short hairpin RNA (shRNA) [13]. Unfortunately, there were no data on the levels of VDRn [13] that could have helped clarify the basis for the remnant 1,25D-stimulated PKC activity that was still evident in these shRNA-treated cells. Further work is, therefore, warranted to ascertain whether the VDRn is coupled to PKC activation using more conventional assays.

Investigations into the mechanisms by which VDRm and VDRn initiate their actions have revealed that, apart from its nuclear localisation, VDRn is also localized to caveolae-enriched plasma membranes [20,21]. Under confocal microscopy, VDRn has been found at the cell surface, perinuclear area and in the nucleus. In the caveolae of MC3T3-E1 osteoblasts, VDRn has been reported to colocalise with VDRm (Pdia3) and caveolin-1 [21]. Co-immunoprecipitation studies have demonstrated that although VDRn does not bind Pdia3 in these cells, it interacts with caveolin-1. In contrast, in human skin fibroblasts, co-immunoprecipitation studies have revealed that VDRn interacts with ERp57 or Pdia3 [26]. As both Pdia3 and VDRn bind caveolin-1, a functional role for caveolin-1 in the action of 1,25D is therefore likely. Indeed, knocking down the expression of caveolin-1 resulted in the loss of non-genomic (src and PLA2 activation) as well as genomic (cell proliferation and expression of alkaline phosphatase) actions of 1,25D in MC3T3-E1 osteoblasts [21]. These observations imply that 1,25D employs caveolin-1 as an essential scaffold to trigger its actions. Interestingly, Pdia3 and VDRn are physically coupled to different downstream effectors. Thus, Pdia3 was found to bind caveolin-1 and PLA2 activation protein that is required for PLA_2_ activation whereas VDRn was found to bind caveolin-1 and src [21]. Despite this difference, both receptors are essential for the activation of src and PLA_2_ since shRNA-mediated knocked down of either Pdia3 or VDRn abrogated the activation of both src and PLA_2_ [21]. Studies in MC3T3-E1 osteoblasts have also demonstrated that upon stimulation with 1,25D, VDRn moves from the caveolae to the nucleus but no such movement is evident for Pdia3 [21]. This shift would enable VDRn to execute its genomic function in the presence of 1,25D.

## 5. Regulation of 1,25D-Mediated Gene Transcription by Non-Genomic Actions

An interesting question which arose following the acceptance of the rapid non-genomic actions was whether these could modulate the genomic function of 1,25D. Indeed, there is strong evidence for this. As discussed above, 1,25D causes the activation of signalling molecules such as MAP kinases, including ERK1/ERK2, ERK5 and JNK1/JNK2, and others such as PKC, PI3K, PLA_2_ and p21ras [12,13,14,15,16,17,27,28]. A range of studies have investigated the roles these play in the genomic function of 1,25D. Studies into the anti-cancer actions of 1,25D have demonstrated that 1,25D induces the differentiation of acute myeloid leukemia cell lines to more mature cells of the monocytic lineage [28]. In these cells, 1,25D up-regulates the activity of ERK1/ERK2 as well as the expression and activation of ERK5. It appears that activation of ERK5 but not ERK1/ERK2 restrains the 1,25D-induced maturation programme since inhibition of ERK5 but not ERK1/ERK2 enabled the cells to mature further along the lineage to display macrophage-like phenotype [28]. This raises the possibility that inhibition of ERK5 would enhance the anti-cancer action of 1,25D and implies that some of the non-genomic action may negatively impact on the action of the secosteroid hormone. However, the role and involvement of the each signalling molecule in mediating the actions of 1,25D may be dependent on the cell-type and/or function under investigation as discussed below.

In growth zone costochondral chondrocytes, ERK1/ERK2 have been reported to be involved in regulating proteoglycan synthesis in response to 1,25D as kinase inhibition resulted in suppression of this response [29]. Inhibition of the ERKs had little or no effect on 1,25D-mediated enhancement of the specific activity of alkaline phosphatase or the inhibition of chondrocyte proliferation. In cells derived from the kidney, the main site for 1,25D inactivation, MAP kinases, such as ERK1/ERK2, JNK1/JNK2 and ERK5, PKC, PI3K and p21ras, have been found to regulate the 1,25D-mediated activation of the CYP24 promoter [14,15,16,30]. These studies have also identified cell-type-specific, gene-specific and maturation stage-specific involvement and roles for these kinases in mediating the actions of 1,25D. In monkey kidney fibroblast COS-1 cells transfected with the rat CYP24 promoter-luciferase construct, Dwivedi *et al*. [14] reported that ERK1/ERK2 and ERK5 were required for 1,25D-mediated activation of the CYP24 promoter since a dominant negative mutant of either ERK1 or MEK5, the upstream regulator of ERK5, attenuated this response; the p38 and JNK kinases were not required. Studies with a dominant negative p21ras mutant revealed that p21ras was upstream of the ERK MAP kinases and required for the genomic action of 1,25D. Overall, the data show that the rapid activation of ERK1/ERK2 and ERK5 is involved in regulating the slower 1,25D-mediated CYP24 promoter activation by phosphorylating RXRα and Ets-1, respectively, thus affecting their ability to act via the vitamin D_3_ response element and the Ets-binding site, respectively, within the CYP24 promoter region [14]. ERK1/ERK2 and ERK5 are also involved in regulating the 1,25D-mediated CYP24 promoter activation and gene expression in Caco 2 enterocyte-like cells [30]. Interestingly, ERK5, by phosphorylating Ets-1, enhanced 1,25D-mediated CYP24 gene transcription only in proliferating but not differentiated Caco-2 cells [30]. An explanation for this is that differentiated cells have reduced levels of ERK5 and Ets-1 protein compared to proliferating cells. Furthermore, in this cell-type, ERK1/ERK2 inhibition did not affect 1,25D-regulated expression of the transient receptor potential cation channel, subfamily V, member 6 (TRPV6) [30]. Through chromatin immunoprecipitation assays and inhibition of ERK1/ERK2, it was found that ERK1/ERK2 targeted Mediator Complex Subunit 1 that was recruited to the CYP24 but not the TRPV6 promoter in response to 1,25D [30]. These data provide evidence of gene-specific and cell stage-specific roles for the ERK1/ERK2 MAP kinases on 1,25D-mediated gene induction in Caco-2 cells.

Selective usage of the MAP kinases by 1,25D on CYP24 promoter activation is observed in human embryonic kidney 293T cells. Nutchey *et al*. [15] reported that 1,25D induction of CYP24 required JNK but not the ERK1/2 in these cells, in contrast to COS-1 cells [14]. Thus, blocking the JNK pathway with a dominant negative mutant of MAP kinase ERK kinase (MKK) 4, an upstream kinase of JNK, caused a reduction in the level of induction whereas a dominant-negative ERK1 mutant had no effect [14]. Consistent with this, 1,25D stimulation resulted in enhanced activity of JNK but not ERK1/ERK2 in these cells. Nevertheless, ERK1/ERK2 was involved in mediating the well-known phenomenon of synergistic up-regulation of CYP24 expression by phorbol 12-myristate 13-acetate (PMA) and 1,25D. Thus, PMA-stimulated activation of ERK1/ERK2 was not only potentiated by 1,25D (Figure 1), which on its own had no effect on kinase activity, but transfection of a dominant-negative ERK1 mutant resulted in a reduced level of synergy on the activity of a CYP24 promoter-luciferase construct [15]. The ERK1/ERK2 pathway has also been reported to underpin this synergy in differentiated Caco-2 cells and the transcription factor, Sp3, appears to be another target of ERK1/ERK2 in mediating this synergy [31] (Figure 1). This is supported by the observations that PMA stimulated the phosphorylation of Sp3 and that inhibition of ERK1/ERK2 not only reduced the degree of synergy between PMA and 1,25D on CYP24 promoter reporter activity but also PMA-stimulated phsophoryation of Sp3 [31]. The role of Sp3 was confirmed by the observation that knockdown of Sp3 reduced the degree of enhancement of CYP24 promoter activity by PMA [31]. The JNK and p38 modules also play a role in supporting this synergy. Thus, it was reported that the duration of JNK activation in response to 1,25D was not only prolonged by PMA treatment but that blocking the JNK module with a dominant negative mutant of MKK4, reduced the degree of synergy [15]. The target of JNK was an as yet unidentified factor that binds to a 5′-TGTCGGTCA-3′ sequence, termed the vitamin D stimulatory element (VSE), located 30 bp upstream of the vitamin D response element-1 in the CYP24 proximal promoter [15] (Figure 1). In differentiated Caco-2 cells, p38 was found to be activated by PMA although the study did not report on the effect of 1,25D on this MAP kinase [31]. Nevertheless, pharmacological inhibition of p38 attenuated both the 1,25D-induction of the CYP24-luciferace reporter construct, as well as the synergy between PMA and 1,25D [31]. Thus, there is cell-type-specific involvement and roles of different MAP kinases on 1,25D-mediated induction of the same gene.

In Madin Darby bovine kidney MDBK epithelial cells, Simboli-Campbell reportedthat 1,25D caused the translocation of PKCα to the plasma membrane and PKCβ to the nucleus, albeit with delayed kinetics compared to those observed when cells are stimulated via other classes of membrane receptors [32]. Other PKC isozymes were unaffected. As PKCβ translocation was accompanied by an increase in nuclear protein phosphorylation, it was proposed that PKCβ was responsible for 1,25D-mediated nuclear events [32]. This is consistent with our observation in HEK293T cells that 1,25D acted via PKCβ1, as well as PKCξ, to induce the activation of the CYP24 promoter as transfection with a dominant negative mutant of either PKC isozyme suppressed 1,25D induction of the CYP24 promoter [15,16]. The data also show that 1,25D rapidly stimulated PI3K activity which was required for both the 1,25D-mediated activation of PKCξ and the PKCξ-dependent phosphorylation of Sp1 that acted via the GC box in the CYP24 promoter region [16]. Overall, these data provide strong evidence for a role of the non-genomic actions in underpinning the genomic actions of 1,25D and reveal some of the mechanisms via which the above-discussed signalling molecules modulate the expression of 1,25D-responsive genes.

## 6. Non-Classical, Non-Genomic Action of the VDR that Involves Protein-Protein Interaction to Regulate Gene Transcription

While the major focus on the non-genomic actions of 1,25D has centred on the signalling pathways that are activated by VDRn and VDRm, there is another mechanism via which, 1,25D, through a non-classical, VDRn-mediated non-genomic action, affects intracellular signalling molecules or transcription factors that influence the expression of a variety of genes. This action may explain some of the modulatory effects that 1,25D exerts on the innate and adaptive immunity, anti-viral responses of cells and cell survival. In general, this involves protein-protein interaction between the VDR and a target protein. Such targets include I-κB kinase (IKK)β, one of the upstream regulators of the canonical NF-κB pathway [33], Signal Transducers and Activators of Transcription (Stat)1 [34], Runt-related transcription factor (RunX)1 [35], c-jun [36], β-catenin [37], cAMP response element-binding protein [38]. Some examples are described below to illustrate this action.

Many studies have shown that 1,25D down-regulates the expression of immune modulatory molecules such as interleukin (IL)-12, IL-8, monocyte chemoattractant protein-1, plasminogen activator inhibitor-1 and angiotensinogen (involved in inflammation associated hypertension), and microRNA-155 (a negative regulator of the expression of suppressor of cytokine signalling 1) by inhibiting NF-κB [39,40,41,42,43]. These effects are unlikely to involve the vitamin D response element. Instead, the liganded VDRn has been reported to down-regulate gene transcription by directly interacting with the key signaling molecule, IKKβ [33]. Such interaction was found to prevent IKKβ phosphorylation on S177/181 that is believed to be needed for IKK signalosome complex formation and the downstream phosphorylation and degradation of I-κBα [33], a prerequisite step in the activation of NF-κB. In T cells, 1,25D is known to suppress a number of T-helper (Th)1 and Th17 functions (reviewed in [35]). Thus, 1,25D has been reported to inhibit the transcription of IL-2, IFN-γ and IL-17A. Whereas inhibition of IFN-γ expression is mediated by a genomic action of VDR/RXR complex that binds to a negative vitamin D response element on the IFN-γ promoter, 1,25D-mediated suppression of IL-2 and IL17 expression is via a blockade of the function of key transcription factors that activate their promoters. For example, 1,25D has been shown to block IL-2 transcription through a process that involves a competitive binding of VDR/RXR to the Nuclear Factor of Activated T cells (NF-AT) element in the IL-2 promoter, thus preventing NFATc1 binding. Although a direct interaction between the VDR or VDR/RXR with NFATc1 has not been demonstrated, the VDR/RXR complex has been shown to prevent the formation of NFATc1/AP1 complex [35]. A similar mechanism is operative in the suppression of IL-17A production by 1,25D [35]. In addition, 1,25D also acts, in part, through a direct sequestration of RunX1 by VDR/RXR that prevents RunX1 from binding to its site on the IL17A promoter [35].

Although metabolites of vitamin D possess anti-hepatitis C virus (HCV) activity [44,45,46], albeit not against some respiratory viruses (reviewed in [47]), 1,25D also enhances the inhibitory effect of IFN-α on HCV replication [34]. This is consistent with the recent findings that vitamin D deficiency is associated with poor responsiveness to pegylated IFN-α and ribavirin therapy in patients with chronic hepatitis C [44,48]. The antiviral activity of IFN-α involves the Janus kinase (Jak)-STAT signalling pathway and activation of JAK1 and Tyk2 results in the phosphorylation of Stat1 and Stat2, transcription factors which are critical for the expression of interferon (IFN)-stimulated genes [34]. In the absence of 1,25D, Lange *et al*. [34] found a constitutive inhibitory interaction between VDRn and Stat1 in a hepatocellular carcinoma cell line. Stimulation with 1,25D and IFN-α released this binding. Although 1,25D did not affect the degree of IFN-α-induced phosphorylation of Stat-1, 1,25D enhanced the IFN-α-induced binding of phosphorylated Stat1 to its IFN-γ activated DNA target sequences and enhanced the expression of IFN-stimulated genes, including IFI27L, IFI44L, ISG15, OAS and RSAD2 [34]. The inhibitory role of VDR was further supported by the observation that silencing of the VDR resulted in an enhanced expression of the above IFN-stimulated genes when the cells were exposed to IFN-α [34]. These data provide a plausible explanation for the poor responsiveness of vitamin D-deficient patients to pegylated IFN-α treatment.

In addition to the above described antiviral actions of 1,25D, the secosteroid hormone also contributes to the innate antimicrobial activity of monocytes/macrophages and other cell types. Data from human monocytes or macrophages have revealed that activation of the cells by ligands of Toll-like receptors 1, 2, or 8, IFN-γ or CD40 ligand, upregulates the expression of CYP27B1 and VDRn (reviewed in [35,49,50]). This results in the bioactivation of 25 hydroxivitamin D to 1,25D by CYP27B1 [50]. Acting via the binding of liganded VDRn to vitamin D_3_ response elements, this autocrine mechanism enables 1,25D to stimulate the transcription and production of anti-microbial peptides such as cathelicidin and human β-defensin 2 (DEFB4) as well as the expression of nucleotide-binding oligomerization domain-containing protein 2 (NOD2), which feeds back to further enhance DEFB4 expression [49,50]. Concomitantly, the liganded VDR mediates downregulation of hepcidin antimicrobial peptide that promotes the export of iron, making the intracellular compartment unfavourable for pathogen survival and proliferation. Furthermore, cathelicidin promotes autophagy, which would enhance autophagolysosomal fusion and antimicrobial activity [49]. In primary human keratinocytes, although 1,25D (10^−8^ M) *per se* has been reported to be sufficient to induce cathelicidin expression, such expression was found to be enhanced by interleukin-17A, which alone is unable to stimulate cathelicidin expression [51]. The synergy between vitamin D metabolites and receptors of the innate and adaptive immune systems has been implicated in the killing of *Pseudomonas aeruginosa* and *Bordetella bronchiseptica* in bronchial epithelial cells [52], killing of intracellular *Mycobacterium tuberculosis* by human macrophages and inhibition of HIV replication in macrophages [53,54], to name a few examples. There is some evidence that a non-genomic action is involved in the induction of the antimicrobial peptides. In keratinocytes, it has been reported that not only was the activity of ERK1/ERK2 enhanced by either IL17A or 1,25D, with further enhancement when the two agents were present together, but that the induction of cathelicidin was also suppressed by more than 50% when the ERK1/ERK2 pathway was inhibited by the pharmacological inhibitor, PD98059 [51]. This again reinforces the premise that genomic actions that involve the vitamin D3 response elements are modulated by the non-genomic actions.

Although vitamin D possesses anti-cancer properties and 1,25D has been reported to promote the differentiation or death of cancer cells ([1,27,28,36] and references therein), there is evidence that also supports an anti-apoptotic function of the VDR, independently of 1,25D. For example, treatment of human breast cancer MDA-MB-468 cells with stressors such as arsenite has been reported to result in cell death [36]. Such treatment of cancer cells also induces the activation of p38 and JNK that results in an upregulation of VDR expression [36,55,56]. One of the consequences of this elevated levels of VDR is the suppression of cell death induced by arsenite, and 1,25D is not required for this action [36,55]. Support for an anti-death function of the VDR comes from the observation that stable transfection of cancer cells with VDR confers resistance against arsenite-induced cell death [36]. Although c-jun, by binding to a c-jun/AP1 site on the VDR promoter, is required for the arsenite-induced upregulation of VDR expression [56], the VDR, in turn, suppresses a c-jun-dependent cell death pathway initiated by arsenite [36]. This non-classical action of the VDR is likely to be mediated by its binding to c-jun [36]. Interestingly, while arsenite increases the degree of this interaction, 1,25D does not affect this phenomenon. The above examples demonstrate that the VDR can exhibit non-classical, non-genomic actions though its ability to interact with various target proteins in the cell.

## 7. Effects of Vitamin D Supplementation on Intracellular Signalling

While the above discussed data were derived from *in vitro* studies using primary cells or cell-lines to which 1,25D was added into the incubation medium, there is some recent evidence that administration of cholecalciferol (vitamin D_3_) to animals can result in the modulation of cellular function and intracellular signalling processes. Consistent with the *in vitro* data that 1,25D suppresses inflammatory cytokine production and inhibits NF-κB mediated gene transcription [33,39,40,41,42,43], it has been reported that dietary supplementation of carp with cholecalciferol suppressed lipopolysaccharide-induced production of inflammatory markers, including tumour necrosis factor (TNF)α, IL-1β, IL-6 and IL-8 and this was likely to have been the consequence of a vitamin D-mediated downregulation of Toll-like receptor (TLR) 4, Myeloid differentiation primary response gene (Myd) 88 and NF-κB p65 mRNA expression [57]. In another study, Al-Rasheed *et al*. [58] reported that dietary supplementation of rats with cholecalciferol suppressed cardiac hypertrophy by down regulating TNF-α production and signalling via the NF-κB pathway as evidenced by a reduction in p65 expression but increased expression of the inhibitory I-κBα at the mRNA level. An inhibitory effect of dietary supplementation with cholecalciferol on the NF-κB pathway, muscle tissue damage and inflammatory response has also been reported in rats that had been subjected to high intensity exercise [59]. In this study, although the effect of 1,25D *per se* on the activities of ERK1/ERK2, p38 and AMP-activated protein kinase (AMPK) was not examined, dietary supplementation with cholecalciferol prevented high intensity exercise from increasing the activities of these kinases as well as inhibiting the activation of NF-κB. In contrast, another study in a model of polycystic ovary syndrome has found that feeding mice with 1,25D prevented the development of cardiac abnormalities and myocardial apoptosis/dysfunction and this was associated with a normalisation of AMPK activity in the myocardium [60]. These studies imply that 1,25D executes non-genomic actions *in vivo*. Currently, there are very limited data in humans in which tissues/cells have been examined for non-genomic actions of vitamins D following dietary supplementation. However, there is one study comparing controls with type 1 diabetes patients in which the authors found that low plasma 25D levels in patients were associated with increased inflammatory markers. Thus, the patients have higher plasma C-reactive protein and higher expression of TLR2/4 and NK-κB activity in their monocytes than controls [61]. It remains to be determined whether increasing the levels of 25D in these patients would reduce the levels of these inflammatory markers to demonstrate a causal relationship. Nevertheless, these data support the idea that the beneficial effects of vitamin D *in vivo* are mediated, at least in part, via a non-genomic action on intracellular signalling. Additional *in vivo* studies are warranted to determine whether 1,25D *per se* exerts the same effects on the intracellular signalling molecules/pathways that had been investigated under *in vitro* conditions. Furthermore, it is also unclear whether blocking the activities of the kinases/pathways can prevent the secosteroid hormone from exerting its effects *in vivo*. A greater understanding of how 1,25D acts *in vivo* would not only open avenues to modulate the actions of 1,25D for therapeutic purposes but also enhance confidence in the use of vitamin D3 as dietary supplements or for nutraceutical purposes. For example, understanding the actions of 1,25D at the signalling level *in vivo* would enable its actions to be pharmacologically manipulated. This will be relevant to situations when CYP27B1 is over-expressed in disease-activated macrophages that results in high levels of 1,25D and increased risk of hypercalcemia as seen in sarcoidosis [62], or when vitamin D-mediated antibacterial responses in monocytes in lepromatous leprosy are corrupted by IL4-induced excessive production of CYP24 [49] that inactivates 1,25D. Similarly, the more classical actions of 1,25D on Ca^2+^ and phosphate homeostasis and bone remodelling, cellular proliferation and differentiation and immune regulation [7,8,9,10] could be manipulated, based on this understanding.

In summary, 1,25D has come a long way from its originally reported role in Ca^2+^ and phosphate homeostasis and its deficiency in the pathogenesis of rickets in children and osteomalacia in adults. At the same time, its mode of action has deviated from one that is reliant on just the VDRn to effect its genomic function to the acceptance of a VDRm and a role of non-genomic actions in regulating a wide range of 1,25D-mediated genomic and cellular functions that impact on general physiological processes, including bone remodelling, cellular proliferation and differentiation, and others such as immune regulation, and anti-cancer and anti-viral/bacterial activities. Several modes of action downstream of VDR ligation by the secosteroid hormone have been identified. However, to fully exploit the potential of 1,25D as a wide-spectrum regulator of human physiology, defining an appropriate minimal level and achieving this level in the body, either through diet or sun exposure, remains a challenge in sections of the population. A better understanding of the non-genomic actions of 1,25D may provide avenues to modulate its actions, particularly in those who are vitamin D-deficient.

## Abbreviations

The following abbreviations are used in this manuscript:

RDA	Recommended Dietary Allowance
25D	25-hydroxyvitamin D
1,25D	1,25 dihydroxyvitamin D
VDRm/n	Membrane or nuclear vitamin D receptor
CYP27B1	ytochrome P450C1α
CYP24	Cytochrome P450C24
RXR	Retinoid-X-Receptor
PLA2	Phospholipase A2
PI3K	Phosphatidylinositol 3-kinase
MAP kinase	Mitogen-activated protein kinase
PKC	Protein kinase C
MARRS	Membrane-associated rapid response steroid
GRP58	Glucose responsive protein, 58 kDa
ERp57 and ERp60	Endoplasmic reticulum protein 57/60
Pdia3	Protein Disulfide Isomerase Family A, Member 3
RNAi	RNA interference
Akt	Protein kinase B
shRNA	Short hairpin RNA
ERK	Extracellular signal Regulated protein Kinase
JNK	c-Jun N-terminal Kinase
TRPV6	Transient receptor potential cation channel, subfamily V, member 6
MKK	MAP kinase ERK kinase
PMA	Phorbol 12-myristate 13-acetate
VSE	vitamin D stimulatory element
IL	Interleukin
IKK	I-κB kinase
Th	T-helper
NFAT	Nuclear factor of activated T cells
RunX	Runt-related transcription factor
AP1	Activator protein 1
IFN	Interferon
HCV	Hepatitis C virus
IFI27L	Interferon, Alpha-Inducible Protein 27 like
IFI44L	Interferon-Induced Protein 44-Like
ISG15	Interferon-Stimulated Protein, 15 KDa
OAS	2′-5′-Oligoadenylate Synthetase
RSAD2	Radical S-Adenosyl Methionine Domain Containing 2
AMPK	AMP-activated protein kinase
TLR	Toll-like receptor
DEFB4	Human β-defensin 2
TNF	Tumour necrosis factor
